# Immunity induced by valine-glycine repeat protein G imparts histoprotection of vital body organs against *Acinetobacter baumannii*

**DOI:** 10.1186/s43141-022-00325-4

**Published:** 2022-03-07

**Authors:** Saeed Alipouri, Iraj Rasooli, Mohammad Hossein Ghaini, Abolfazl Jahangiri, Shakiba Darvish Alipour Astaneh, Fatemeh Ramezanalizadeh

**Affiliations:** 1 Department of Biology, Shahed University, Qom Expressway, Tehran, 3319118651 Iran; 2 Molecular Microbiology Research Center and Department of Biology, Shahed University, Tehran, Iran; 3 Department of Anatomical Sciences and Pathology, School of Medicine, Shahed University, Tehran, Iran; 4 Applied Microbiology Research Center, Systems Biology and Poisonings Institute, Baqiyatallah University of Medical Sciences, Tehran, Iran; 5 Department of Biotechnology, Semnan University, Central Administration of Semnan University, Campus 1, Semnan, I. R. of Iran Semnan, P.O. Box 35131-19111, Semnan, Iran; 6 Department of Microbiology, Shahed University, Tehran, Iran

**Keywords:** *Acinetobacter baumannii*, Vaccine, Immunity, Active immunization, Passive immunization, VgrG

## Abstract

**Background:**

Efforts toward the development of an effective vaccine against *Acinetobacter baumannii*, one of the most notorious nosocomial pathogens, are still ongoing. In this regard, virulence factors are interesting targets. Type VI secretion system (T6SS) participates in the pathogenicity of *A. baumannii*. VgrG is a crucial component of T6SS prevalent among *A. baumannii* strains. This study was conducted to evaluate the immunoprotectivity of recombinant VgrG (rVgrG) cloned and over-expressed in *Escherichia coli* BL21 (DE3). BALB/c mice were immunized with the purified rVgrG. Specific anti-VgrG IgG titers were assessed by ELISA. Actively and passively immunized mice were challenged with lethal doses of *A. baumannii* ATCC 19606. The survival rate, the bacterial burden, and histopathology of tissues in infected mice were examined.

**Results:**

Anti-VgrG IgG (*p* < 0.0001) was significantly increased in immunized mice. No death was seen in actively immunized mice infected with the lethal dose (LD) of 1.9 × 10^8^ CFU of *A. baumannii* ATCC 19606 within 72 h. Challenge with 2.4 × 10^8^ CFU of the pathogen showed a 75% survival rate. All immunized mice infected with 3.2 × 10^8^ CFU of the pathogen died within 12 h. In passive immunization, no death was observed in mice that received LD of the bacteria incubated with the 1:250 dilution of the immune sera. An increased number of neutrophils around the peribronchial and perivascular areas were seen in unimmunized mouse lungs while passively immunized mice revealed moderate inflammation with infiltration of mixed mononuclear cells and neutrophils. The livers of the unimmunized mice showed inflammation and necrosis in contrast to the livers from immunized mice. Hyperplasia of the white pulp and higher neutrophils were evident in the spleen of unimmunized mice as against the normal histology of the immunized group.

**Conclusions:**

VgrG is a protective antigen that could be topologically accessible to the host antibodies. Although VgrG is not sufficient to be assigned as a stand-alone antigen for conferring full protection, it could participate in multivalent vaccine developments for elevated efficacy.

**Supplementary Information:**

The online version contains supplementary material available at 10.1186/s43141-022-00325-4.

## Background

*Acinetobacter baumannii*, a successful nosocomial pathogen, is a serious health threat such that the Infectious Diseases Society of America (IDSA) assigned the pathogen as one of the six most dangerous microbes [1]. Moreover, based on World Health Organization (WHO) ranking, this is the 1st pathogen that urgently needs new antibiotics. However, no effective approved antibiotic is introduced against drug-resistant strains of *A. baumannii*. Hence, active and passive immunizations could be invoked as an alternative solution against the notorious pathogen. Several studies nominated promising immunogens for active and passive immunizations against *A. baumannii* [1–19]. However, none has been investigated in clinical trials. Animal studies have revealed that perfect protection could not be achieved by the administration of a single antigen. In this regard, investigations about *A. baumannii* antigens are still ongoing. Virulence factors are among the most attractive targets for immunization against pathogens. Secretion systems have pivotal roles in the pathogenicity of bacteria. Type VI secretion system (T6SS), a weapon in many Gram-negative bacteria, is responsible for delivering many toxic protein effectors into prokaryotic and eukaryotic prey cells [20]. This apparatus comprised at least 13 conserved proteins consisting of a baseplate, a membrane-spanning structure, a contractile sheath, a cytoplasmic sheath recycling protein, and an injectable needle. However, various bacteria could produce their accessory proteins and secretory effectors. The hemolysin co-regulated protein (Hcp), the proline-alanine-alanine-arginine repeat (PAAR) protein, and a trimer of valine-glycine-arginine G (VgrG) are involved in the formation of the injectable needle in which Hcp hexamers are from the main tube. The tube is capped with VgrG and, in some cases, the PAAR protein [20]. T6SS has also been identified in many strains of *A. baumannii* [21]. However, as a difference, it has no homolog for a conserved outer membrane lipoprotein, TssJ, of *Escherichia coli* [21]. Previous studies unveiled that, among components of T6SS, VgrG is an appropriate candidate for the investigation of active and passive immunizations. This protein increases *A. baumannii* virulence and its adhesion to lung epithelial cells [22]. In silico analyses revealed that VgrG is present in most *A. baumannii* strains [23, 24]; T6SS-positive strains usually harbor 2–4 copies of the *vgrG* gene [23]. The C terminus of this crucial structural component of T6SS is essential for the functional assembly of T6SS nanomachine in *A. baumannii* [25]. Hence, VgrG is an impressive virulence factor to be explored with immunization. Recently, we studied the VgrG_421-765_ and VgrG_421-536_ regions regarding immunoprotective effects. Challenge with a lethal dose of *A. baumannii* ATCC 19606 in mice immunized with VgrG_421-765_ and VgrG_421-536_ showed 33% and 66% survival rates, respectively [2]. Surprisingly, VgrG_421-536_ developed higher protectivity; however, none of the selected regions conferred full protection against a lethal dose of the bacteria. Although immunoprotective effects of the selected regions were elucidated, protective effects of VgrG encompassing both N- and C-terminal domains remained to be addressed. The current study is conducted to evaluate the immunoprotective efficacy of the recombinant VgrG in a murine model.

## Methods

### Overexpression of VgrG

In the current study, genomic DNA of *A. baumannii* ATCC 19606 was used as a source of the *vgrG* gene. A pair of primers (forward: CAAGGATCCATGGTATTCTTACAACGTATAGAAGGCCAACATC and reverse: CCGTCTAGATTACATACATTCTTGCTCCATCTTGAGCTGCAA) was designed to amplify the *vgrG* gene by polymerase chain reaction (PCR). The primers were harboring restriction sites of *Bam*HI and *Xba*I enzymes (underlined). The PCR product and the expression plasmid (e5044) were digested with *Bam*HI and *Xba*I. The digested amplicon was ligated to the plasmid and transformed to competent cells of *Escherichia coli* BL21 (DE3) strain. The transformants were grown overnight at 37 °C in an auto-induction medium [26] supplemented with 100 μg/mL ampicillin. The cells were harvested by centrifugation and resuspended in denaturing buffer (8 M urea, 10 mM Tris-HCl, 100 mM NaH_2_PO_4_, pH 8). The cell suspension was sonicated and then was centrifuged at 13000 rpm, for 20 min at 4 °C. The supernatant was used for the purification procedure. The recombinant protein was purified in denaturing conditions by the Ni-NTA affinity column (Qiagen, Germany). The purified protein was analyzed by 9% sodium dodecyl sulfate-polyacrylamide gel electrophoresis (SDS-PAGE). The denatured protein was refolded by sequential dialysis against PBS containing 6, 4, 2, and 0 M urea + 0.5 mM l-arginine (pH 7.4) at 4 °C for 2 h. Bradford protein assay [27] was used to estimate the concentration of the purified recombinant protein.

### Mouse immunization

Twenty-four 6- to 8-week-old female BALB/c mice weighing 20–25 g were distributed into two groups of 12 mice each in the control group and the test groups. In the first injection, the test group received subcutaneously 20 μg of the purified recombinant protein mixed with 1:1 (v/v) ratio of complete Freund’s adjuvant (Sigma-Aldrich, Merck KGaA, Germany). Boosters were administered on days 15 and 30 with 20 μg of the refolded recombinant protein admixed with incomplete Freund’s adjuvant (Sigma-Aldrich) at a 1:1 (v/v) ratio. The control group received PBS mixed with 0.5 mg arginine and emulsified with a 1:1 (v/v) ratio of the adjuvants. The mice received 100 μL of the prepared mixture in each injection. Blood samples of mice were collected on days 14, 29, and 44. The sera were separated and stored at 20 °C.

### Enzyme-linked immunosorbent assay (ELISA)

Anti-VgrG-specific IgGs in the sera collected from the immunized mice were assessed by indirect ELISA. Briefly, the recombinant VgrG (2 μg/well) was coated in a 96-well ELISA plate. After incubation (overnight at 4 °C), the wells were washed three times with PBST (PBS containing 0.05% Tween 20), and then 100 μL of blocking solution (5% skimmed milk in PBST) was added to the wells. The plate was incubated at 37 °C for 1 h followed by washing (3 times with PBST), after which 100 μL of serially diluted (1:250 to 1:64000) sera was added to the wells. The plate was incubated at 37 °C for 2 h, and then the washing step was repeated. A secondary antibody (horseradish peroxidase-conjugated antibody), diluted to 1:15000 in PBST, was added (100 μL/well). The plate was incubated at 37 °C for 1 h followed by a washing step. Then, 3,3-5,5-tetramethylbenzidine (TMB) solution was added as a substrate (100 μL/well) to develop color. The reaction was stopped by the addition of 3 M H_2_SO_4_, and the absorbance was read at 450 nm using an ELISA reader. The endpoint titer was defined as the highest dilution at which the optical density was 0.1 greater than that of control wells receiving control adjuvant serum.

### Western blotting

The recombinant protein expression was validated by Western blotting with horseradish peroxidase (HRP)-conjugated anti-polyhistidine antibodies (1:10,000 dilution) in which 0.5 μg of the recombinant protein was loaded onto the SDS-PAGE. The recombinant protein was separated on 9% sodium dodecyl sulfate-polyacrylamide gel electrophoresis and then transferred onto a nitrocellulose membrane using transfer buffer (150 mM glycine, 20 mM Tris-base, and 20% methanol). The membrane was blocked with 5% skim milk in PBST at 4 °C overnight. The membrane was incubated with pooled antiserum samples at a dilution of 1:2000 for 2 h and then incubated with a horseradish peroxidase secondary antibody at a 1:10,000 dilution with gentle shaking for 2 h at room temperature. The membrane was washed three times with PBST, and the immunoblot was developed using 3,3-diaminobenzidine (Sigma).

### Bacterial challenges

#### Actively immunized mouse group

*A. baumannii* ATCC 19606 was employed for challenges with viable bacteria in mice. The lethal dose of *A. baumannii* ATCC 19606 was determined. The control and actively immunized mouse groups received intraperitoneally 1.9 × 10^8^ CFU, 2.4 × 10^8^ CFU, and 3.2 × 10^8^ CFU of *A. baumannii* ATCC 19606. The mouse survival was monitored for 72 h. The survived mice were sacrificed, and their spleens and lungs were removed aseptically. The organs were weighed and homogenized in sterile normal saline. The mixtures were serially diluted and plated on LB agar. The plates were incubated overnight at 37 °C.

#### Passive immunization and histopathology

In passive immunization, inocula for each mouse were prepared as follows: after inactivation of the complement system, the sera (1:250 dilutions) obtained from immune or non-immune mice were incubated with 1.9 × 10^8^ CFU of *A. baumannii* ATCC 19606. The suspensions were incubated in a shaking incubator at 37 °C for 2 h. The bacteria were harvested by centrifugation and re-suspended in 100 μL sterile PBS. The suspensions were intraperitoneally injected into the mice. The mouse survival was monitored for 72 h followed by scarification of the survivals. Their liver, spleen, and lungs were aseptically removed and processed for a histopathology examination. The biopsies were fixed in 10% buffered formalin followed by staining with hematoxylin-eosin. The histopathological changes were observed under a light microscope.

#### Statistical analyses

Statistical analyses were performed using the GraphPad Prism 8.0 software. The data were presented as mean with standard deviations represented as error bars. Comparison of antibody titers was performed using a Kruskal-Wallis test followed by Dunn’s multiple comparison test. The bacterial burdens were compared with an unpaired Student’s *t*-test. Survival was compared using the non-parametric log-rank test. Differences were considered significant if the *p-*value was < 0.05.

## Results

### Recombinant VgrG expression and purification

The *vgrG* gene was successfully amplified by PCR from the *A. baumannii* ATCC 19606 genome. The cloned gene into the e5044 plasmid was confirmed via sequencing. The purified recombinant VgrG expressed in *E. coli* BL21 (DE3) revealed a band with an apparent molecular weight of approximately 126 kDa on 9% SDS-PAGE and confirmed by Western blot analysis (Fig. S1).

### Anti-VgrG immunoglobulin G (IgG)

**Fig. 1:**
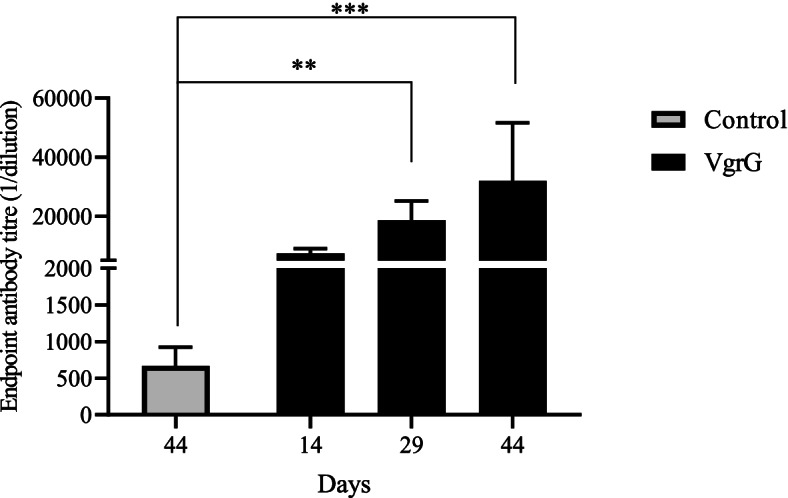
IgG titers determined by indirect ELISA against the recombinant VgrG. The endpoint titer was defined as the highest dilution at which the optical density was 0.1 greater than that of the control wells receiving control adjuvant serum. Comparisons between the groups were performed using a Kruskal-Wallis test followed by Dunn’s multiple comparison test (**p < 01, ***p < 001)

ELISA data (Fig. 1) indicated that the levels of IgG raised to VgrG increased significantly after the second immunization (mean value = 18000) (*p <*0.01). Antibody titers were higher after the third immunization with a mean value of 32,000; the difference was significant compared to the control group (*p* < 0.001).

### Active immunization

**Fig. 2:**
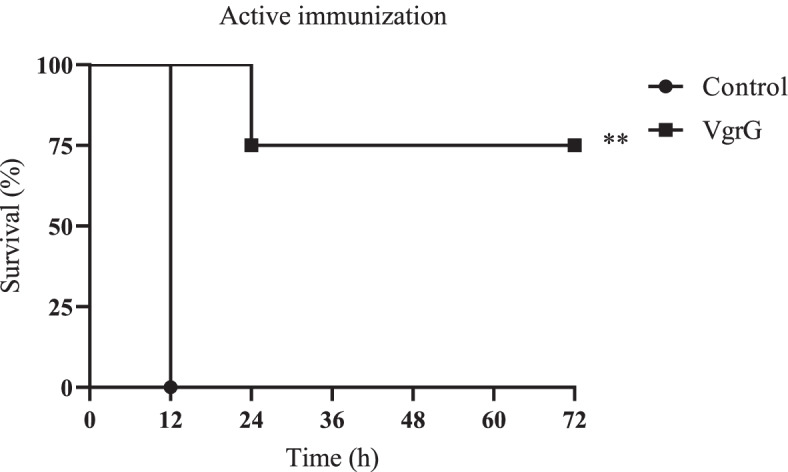
The survival rate in actively immunized mice. The non-parametric log-rank test was used for the analyses of survival rates (*p* < 0.001)

**Fig. 3:**
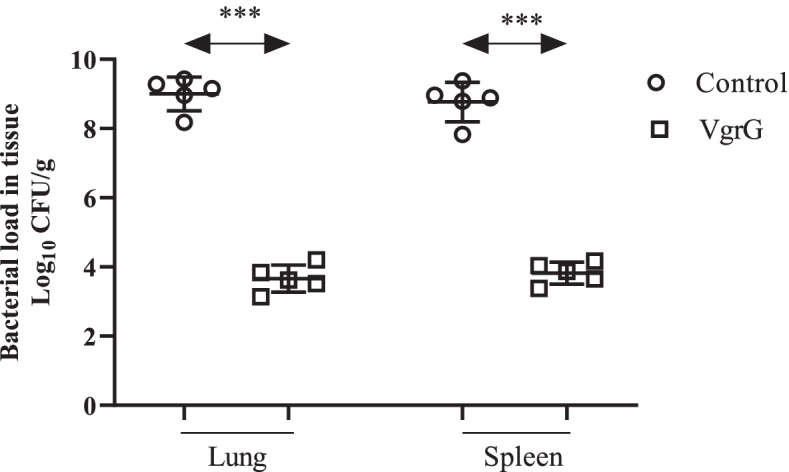
Bacterial burden in the lungs and spleens of the control and actively immunized mouse groups

LD_50_ and LD of *A. baumannii* ATCC 19606 were determined as 1.3 × 10^8^ CFU and 1.9 × 10^8^ CFU, respectively. All control mice that received 1.9 × 10^8^ CFU (LD), 2.4 × 10^8^ CFU, and 3.2 × 10^8^ CFU of *A. baumannii* ATCC 19606 died within 12 h. No death was seen in the test group challenged with the lethal dose of *A. baumannii* ATCC 19606. In the test group that received 2.4 × 10^8^ CFU of *A. baumannii* ATCC 19606, 75% of mice survived within 72 h (Fig. 2). All the immunized mice that received 3.2 × 10^8^ CFU of *A. baumannii* ATCC 19606 died within 12 h (Fig. 2). Immunization with the recombinant VgrG significantly (*p* < 0.001) reduced the bacterial burden in the lung and spleen by approximately 4–5 log cycles 12 h postinfection as compared to adjuvant control (Fig. 3).

### Passive immunization

**Fig. 4:**
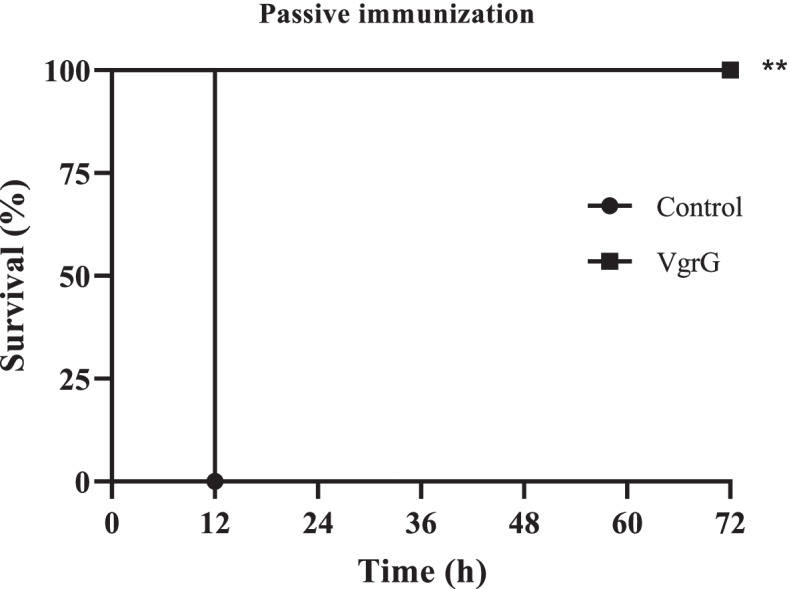
The mouse survival rate in passive immunization. The non-parametric log-rank test was used for the analyses of survival rates (*p* < 0.001)

In passive immunization, 1.9 × 10^8^ CFU of *A. baumannii* ATCC 19606 incubated with the sera (1:250 dilutions) obtained from immune or non-immune mice was used as inoculums for each mouse. All control mice died within 12 h while no death was seen in the test group within 72 h (Fig. 4).

### Histopathology

**Fig. 5:**
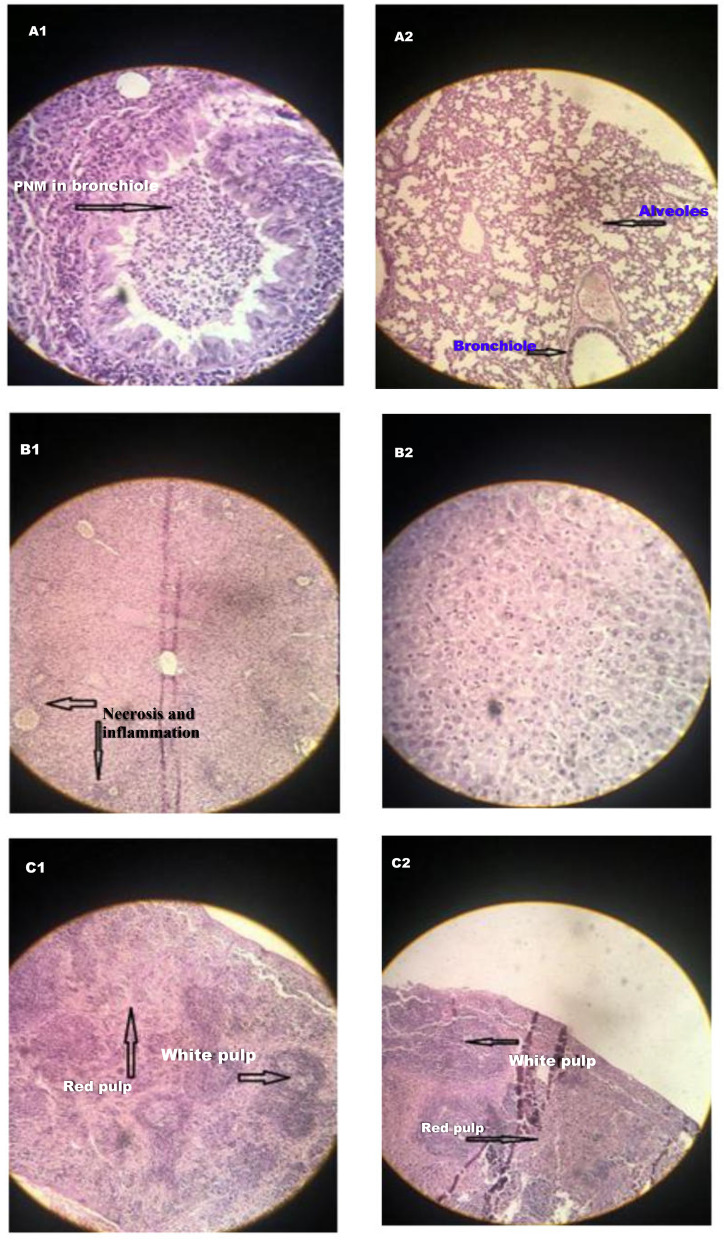
Histopathological examinations of the lung (**A1, A2**), liver (**B1, B2**), and spleen (**C1, C2**) in unimmunized and passively immunized mice challenged with *A. baumannii*. The bacterial challenge caused an increasing number of neutrophils around the peribronchial and perivascular areas in unimmunized mouse lungs (**A1**) while immunized mice revealed moderate inflammation with infiltration of mixed mononuclear cells and neutrophils (**A2**). The livers of unimmunized mice show inflammation and necrosis (**B1**). In contrast, the livers from immunized mice showed no significant pathology (**B2**). The spleen of unimmunized mice showed hyperplasia of the white pulp and higher neutrophils (**C1**) as against the normal structure retained in the immunized group (**C2**)

Histopathological examinations showed that bacterial challenge caused an increasing number of neutrophils around the peribronchial and perivascular areas in unimmunized mice while immunized mice revealed moderate inflammation with infiltration of mixed mononuclear cells and neutrophils (Fig. 5). Passive immunization with anti-VgrG sera reduced liver pathology following challenge with the pathogen. The livers of unimmunized mice show inflammation and necrosis. In contrast, the livers from immunized mice showed no significant histopathologic changes (Fig. 5). The spleen of unimmunized mice showed hyperplasia of the white pulp and higher neutrophils (Fig. 5).

## Discussion

Secretion systems play pivotal roles in the pathogenicity of bacteria. VgrG is a component of T6SS that could increase *A. baumannii* virulence and its adhesion to lung epithelial cells [22]. This virulence factor is present in most *A. baumannii* strains [23, 24]. VgrG is located at the tip of the injectable needle of T6SS; hence, it could be exposed to host antibodies. The recombinant protein encompassing both N- and C-terminal domains include most of the potential epitopes. In our recent study, mice immunized with VgrG_421-765_ showed a 33% survival rate in a challenge with LD of *A. baumannii* ATCC 19606 [2]. In the present study, overexpression of the recombinant VgrG resulted in the formation of inclusion bodies. So, the protein was purified in denaturing conditions. The purified VgrG was aggregated during dialysis against PBS buffer. Hence, l-arginine was added to prevent aggregate formation [5]. Full protection was observed in the immunized mice against a lethal dose of *A. baumannii* demonstrating that the N terminus of VgrG contains important epitopes which could elevate the immunoprotective efficacy of VgrG. The conferred protection revealed that VgrG is expressed by the pathogen in the murine sepsis model. Since T6SS is energetically costly, many T6SS-positive strains tightly regulate its expression at various levels. In *A. baumannii*, some strains have a constitutively active T6SS which also express under standard laboratory conditions; some strains possess a silenced T6SS transcriptionally repressed by large conjugative plasmids (LCPs) of multidrug-resistance; the last strains are those regulating their T6SS by unknown mechanisms [25]. The immunoprotective efficacy of vaccination could be affected by the expression level of VgrG at the infection condition. Some antigens are associated with specific infection types of *A. baumannii* [28]. In contrast, some antigens are associated with different infection types [28]. Immunization with VacJ, a 299 amino acid lipoprotein, showed no significant protection against LD_50_ of *A. baumannii* ATCC 19606 in a murine sepsis model. However, in a murine pneumonia model, immunized mice showed a 600-fold reduction of the bacterial load in the lungs [29]. Protectivity levels of various antigens vary from one antigen to the other. rBauA could confer full protection against 100× LD_50_ of *A. baumannii* ATCC 19606 in actively immunized mice [30]. Mice actively immunized with Bap had shown full protection against 100× LD_50_ of a clinical strain of *A. baumannii* [31]. The survival rate of Bap-immunized mice challenged with 10^5^× LD_50_ of the clinical strain was 60% [31]. Mice immunized with FimA had revealed full protection against 10× LD_50_ of *A. baumannii* ATCC 19606 [32]. The present study demonstrated that VgrG could not develop full protection against more than minimal lethal doses of *A. baumannii* ATCC 19606. This dose is < 2× LD_50_ of the pathogen. Administration dose of the antigen is an important criterion in immune responses and immunoprotective efficacy. It has been shown that, in a sepsis model, immunization of mice with 50 μg and 20 μg of Omp22 could provide 100% and 33% survival against a lethal dose of a clinical strain, respectively [1]. In our previous study, mice who received 40 μg of VacJ showed higher titers of specific antibodies in comparison with mice who received 20 μg of the antigen such that in mice received 40 μg of VacJ, 3 injections were sufficient to trigger high titer of anti-VacJ antibodies [29]. Although only the dose of 20 μg VgrG was investigated in the present study, it would be expected that higher administration doses could increase immune responses and immunoprotective efficacy. Since VgrG is located at the tip of T6SS, it is highly accessible to host antibodies. It has been demonstrated that anti-OmpA monoclonal antibodies (MAbs) could not confer protection against encapsulated strains of *A. baumannii*, in a murine sepsis model, owing to shielding of OmpA by capsule polysaccharide which could inhibit binding of anti-OmpA MAbs to the bacteria [33]. Anti-VgrG antibodies could overcome this obstacle because VgrG is topologically more accessible than OmpA.

## Conclusion

VgrG is found to be a protective antigen to take a part in vaccine developments. This antigen could be topologically accessible to host antibodies even in encapsulated strains of *A. baumannii*. However, some considerations such as species specificity need to be considered.

## Supplementary Information

**Additional file 1: Figure S1**. Expression (a) and western blotting (b) of the recombinant VgrG. a. Expression of recombinant VgrG. The supernatant of the lysed cells in denaturing buffer (buffer B) showing expression of the  ~ 126 kDa recombinant VgrG. b. Confirmation of the recombinant VgrG by Western blotting.

## Data Availability

The datasets generated and/or analyzed during the current study are available from the corresponding author on reasonable request.
