# Supplementary figures and images for: Immunity induced by valine-glycine repeat protein G imparts histoprotection of vital body organs against Acinetobacter baumannii
Source: J Genet Eng Biotechnol. 2022 Mar 7;20:42. doi: 10.1186/s43141-022-00325-4 (PMC8901899; doi:10.1186/s43141-022-00325-4)

| 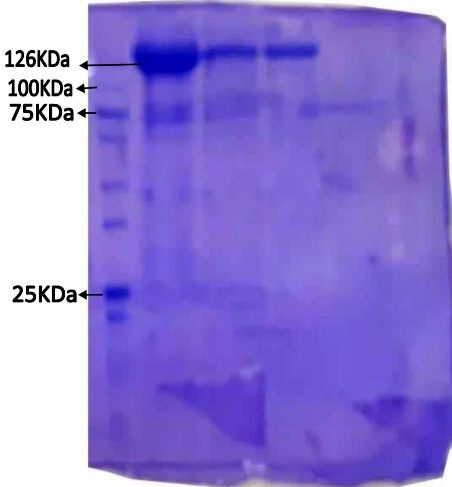**a** | 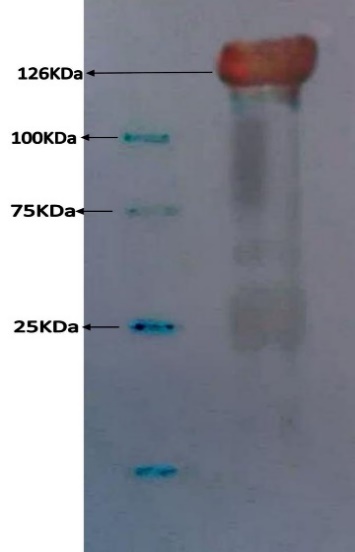**b** |
| --- | --- |

Supplement: Supplementary file 1 — Additional file 1: Figure S1. Expression (a) and western blotting (b) of the recombinant VgrG. a. Expression of recombinant VgrG. The supernatant of the lysed cells in denaturing buffer (buffer B) showing expression of the ~ 126 kDa recombinant VgrG. b. Confirmation of the recombinant VgrG by Western blotting. [file 43141_2022_325_MOESM1_ESM.docx]
